# Comment on Aldén et al. Intracellular Reverse Transcription of Pfizer BioNTech COVID-19 mRNA Vaccine BNT162b2 In Vitro in Human Liver Cell Line. *Curr. Issues Mol. Biol*. 2022, *44*, 1115–1126

**DOI:** 10.3390/cimb44040113

**Published:** 2022-04-11

**Authors:** Hamid A. Merchant

**Affiliations:** Department of Pharmacy, School of Applied Sciences, University of Huddersfield, Queensgate, Huddersfield HD1 3DH, West Yorkshire, UK; hamid.merchant@hud.ac.uk; Tel.: +44-1484-256958

Aldén et al. (2022) recently reported the intracellular reverse transcription of Pfizer BioNTech COVID-19 mRNA Vaccine BNT162b2 in vitro in a human liver cell line (Huh7) [1] that has raised significant concerns over the consequential genotoxicity among mRNA-vaccinated subjects.

The novel COVID-19 vaccines have been subject to several controversies since the beginning, and concerns over their potential to be incorporated into the human genome or alter human DNA (genotoxicity) have been a major public concern, also exploited by anti-vaccine campaigners, and have significantly affected vaccine uptake and corroborated to vaccine hesitancy globally. This article explains reasons as to why such a phenomenon, demonstrated recently in vitro, may not manifest clinically in vivo and therefore cannot be generalised to the healthy population.

First, although Huh7 responds to INF stimulation and is a promising cell line for studying viral infection and replication in vitro [2], it does not reflect an in vivo environment, particularly the absence of comprehensive cellular and humoral immune response. The experimental model employed by Aldén et al. [1] is scientifically incompetent to evaluate the genotoxicity of mRNA therapeutics, including BNT162b2 COVID-19 vaccines. The vaccine distribution beyond the injection site and consequent transfection to hepatocytes, albeit a possibility [3,4], will result in the translation of the mRNA into spike proteins that will attract an immune response towards vaccine-transfected hepatocytes. In the majority of cases, a healthy immune response, mediated by cytotoxic T cells and anti-spike antibodies, will eventually clear off vaccine-transfected hepatocytes; therefore, the reverse transcription of mRNA may not be a reality in vivo. 

Second, the vaccine dose used in vitro is much higher than expected in vivo. The authors argue that the 0.5 to 2 μg/mL vaccine concentrations used in their in vitro experiments are reflective of the in vivo distribution of vaccine in hepatocytes. The cultured cell density and volume used under in vitro setting is way below the in vivo hepatic distribution volume fraction in a human subject. Moreover, the concentration calculations are based on an 18% hepatic distribution of the vaccine from pharmacokinetics studies cited in the EMA report [5]. The pharmacokinetic studies in question refer to the distribution of structural lipids in hepatocytes and not the mRNA itself. After offloading encapsulated mRNA, the disentangled lipids from the vaccine formulation are expected to be distributed and cleared by the liver. The distribution fractions reported in an EMA report [5] cannot distinguish the distribution of fragmented nanoparticles (disentangled lipids) from mRNA-encapsulated intact nanoparticles due to the limitations of the employed assay methods. 

Third, the experiment employed cultured hepatocellular carcinoma cells (Huh-7) that differ significantly from primary human hepatocytes. Authors also acknowledged in their article that Huh-7 exhibits an active DNA replication and significantly different gene and protein expression to the healthy hepatocytes. In particular, the upregulated proteins in Huh-7 involvement in RNA metabolism may favour reverse transcription of the in vitro-transfected mRNA, which may not otherwise occur in a healthy liver in vivo. A good review of mRNA pharmacology and cellular uptake from naked vs. formulated mRNA products can be found in [6]. 

Fourth, retroviruses in particular are known to reverse-transcribe intracellularly and have the ability to be integrated into the host genome. There is some evidence in support of SARS-CoV-2’s ability to integrate some of its genetic sequences into the DNA of the host cells [7]; however, unlike retroviruses, the infectious SARS-CoV-2 virus could not be reproduced from the integrated subgenomic sequences. This evidence is still inconclusive but may explain the prolonged detection of non-infectious virus by a positive PCR test in convalescent patients. This might also hint at the mechanisms behind ‘long-COVID’ observed in a significant number of COVID-19 patients. Ancient viruses have been lurking in the human genome for some time since their integration into our ancestral genome a long ago [8]. Human endogenous retroviruses (HERVs) may constitute 4 to 8% of the total human genome and are believed to be a part of our genetic evolution that offers the fitness gain to species against environmental pathogens. The health implications of carrying viral genomic debris are, however, not fully understood; some may even contribute to diseases such as HIV.

In conclusion, the post-injection mRNA distribution and transfection to hepatocytes is not impossible but will trigger an immune response (cytotoxic T cells and anti-spike antibodies) against the vaccine-transfected hepatocytes. This response is likely to be transient and very specific towards ‘abnormal hepatocytes’, leading to the clearance of transfected hepatocytes by the immune cells; therefore, the reverse transcription of mRNA may not be possible in vivo. The in vitro data presented by Aldén et al. [1] without any in vivo validation in an appropriate animal model (for instance, the transgenic Fischer 344 Big Blue^®^ rats in vivo mutation assay) can lead to misleading inferences. The current findings from Aldén et al. [1] may be detrimental to public confidence in mRNA therapeutics in general if not proven in vivo.

It should be noted that any immune-mediated clearance of hepatocytes may result in reversible functional hepatic and biliary effects (hepatomegaly, vacuolation, increased gamma-GT, AST, and ALP), as seen in animal and clinical evaluation of COVID-19 vaccines and also reported in the EMA assessment report [5]. Some of the hepatic changes may be due to prolonged accumulation of ALC lipids (employed to encapsulate mRNA in vaccine formulation) in the hepatocytes instead of those mediated by the mRNA itself. These hepatic effects were transient and chiefly reversible in preclinical (animal) and clinical evaluation of vaccines. However, the possibility of prolonged liver effects or extended autoimmune response to hepatocytes cannot be overruled in special populations such as those with pre-existing conditions or those who are immunocompromised. COVID-19 vaccines pharmacovigilance has shown many cases of post-immunisation cholestatic and/or autoimmune hepatitis [9], with some clinical case reports with liver histology now being fully published [10,11]. However, this does not prove any causation but warrants further investigations to ascertain the safety of mRNA and/or viral vector vaccines among special populations, such as subjects at high risk of liver injury, those with pre-existing liver conditions, autoimmune disorders of the liver and biliary system (e.g., autoimmune hepatitis or primary biliary cholangitis), hepatocellular carcinoma, or other liver cancers, and importantly, immunocompromised subjects, for instance, those with current or previous HIV, organ transplant, or those on chronic immunosuppressant therapies. Regulatory agencies are requested to investigate post-immunisation pharmacovigilance signals with immunocompromised status of the vaccinated subjects to analyse any potential correlations needing further investigations. Vaccines are a great discovery in medicine that have improved life expectancy dramatically. Nevertheless, genetic vaccines are new, and their comprehensive evaluation in special populations is imperative to reassure vaccine safety in subjects with pre-existing conditions. If any contraindications or safety risks to novel (mRNA- or viral-vector-based) COVID-19 vaccines in special populations are to be established in the future, the COVID-19 vaccine products encompassing non-genetic formulations strategies that are also being deployed across the world, for instance, the whole inactivated virus vaccine (Covaxin, Valneva, Sinopharm, Sinovac) or the recombinant protein COVID-19 vaccines (Novavax, Vidprevtyn), can be promising alternatives for special populations. 
